# Prediction of post-delivery hemoglobin levels with machine learning algorithms

**DOI:** 10.1038/s41598-024-64278-z

**Published:** 2024-06-17

**Authors:** Sepehr Aghajanian, Kyana Jafarabady, Mohammad Abbasi, Fateme Mohammadifard, Mina Bakhshali Bakhtiari, Nasim Shokouhi, Soraya Saleh Gargari, Mahmood Bakhtiyari

**Affiliations:** 1 Student Research Committee, School of Medicine, Alborz University of Medical Sciences, Karaj, Iran; 2 Neuroscience Research Center, Iran University of Medical Sciences, Tehran, Iran; 3 Department of Obstetrics and Gynecology, School of Medicine, Shahid Beheshti University of Medical Sciences, Tehran, Iran; 4 Yas University Hospital, Tehran University of Medical Sciences, Tehran, Iran; 5 Department of Community Medicine, School of Medicine, Alborz University of Medical Sciences, Karaj, Iran; 6 Non-Communicable Diseases Research Center, Alborz University of Medical Sciences, Karaj, Iran; 7 Men′s health and Reproductive health Research Center, Shahid Beheshti University of Medical Sciences, Tehran, Iran

**Keywords:** Postpartum hemorrhage, Machine learning, Multilayer perceptron, Support vector machine, Extreme gradient boosting, Artificial intelligence, Machine learning, Predictive medicine, Medical research, Risk factors

## Abstract

Predicting postpartum hemorrhage (PPH) before delivery is crucial for enhancing patient outcomes, enabling timely transfer and implementation of prophylactic therapies. We attempted to utilize machine learning (ML) using basic pre-labor clinical data and laboratory measurements to predict postpartum Hemoglobin (Hb) in non-complicated singleton pregnancies. The local databases of two academic care centers on patient delivery were incorporated into the current study. Patients with preexisting coagulopathy, traumatic cases, and allogenic blood transfusion were excluded from all analyses. The association of pre-delivery variables with 24-h post-delivery hemoglobin level was evaluated using feature selection with Elastic Net regression and Random Forest algorithms. A suite of ML algorithms was employed to predict post-delivery Hb levels. Out of 2051 pregnant women, 1974 were included in the final analysis. After data pre-processing and redundant variable removal, the top predictors selected via feature selection for predicting post-delivery Hb were parity (B: 0.09 [0.05–0.12]), gestational age, pre-delivery hemoglobin (B:0.83 [0.80–0.85]) and fibrinogen levels (B:0.01 [0.01–0.01]), and pre-labor platelet count (B*1000: 0.77 [0.30–1.23]). Among the trained algorithms, artificial neural network provided the most accurate model (Root mean squared error: 0.62), which was subsequently deployed as a web-based calculator: https://predictivecalculators.shinyapps.io/ANN-HB. The current study shows that ML models could be utilized as accurate predictors of indirect measures of PPH and can be readily incorporated into healthcare systems. Further studies with heterogenous population-based samples may further improve the generalizability of these models.

## Introduction

Postpartum hemorrhage (PPH) is a critical global issue in obstetrics, representing the foremost cause of maternal morbidity and mortality worldwide, contributing to nearly one-third of deaths among pregnant and postpartum women. In the United States, PPH rates are on the rise, complicating almost 3% of deliveries^1^. Recent decades have witnessed advancements in PPH treatment, including compression sutures^2,3^, and changes in fibrinogen and blood transfusion strategies^4,5^. However, the limited availability of these advanced treatments in primary and secondary centers impedes widespread use, underscoring the pivotal role of timely intervention.

Notwithstanding the utilization of advanced therapeutic modalities, postpartum hemorrhage (PPH) continues to exert a pivotal influence on maternal mortality rates. While maternal death is relatively rare in the United States, blood transfusion following hemorrhage, a condition 50 times more prevalent than mortality, is the primary diagnosis linked to severe maternal morbidity^6,7^. The complexity of obstetric care settings, with varying levels of resources and expertise across different healthcare facilities, presents additional challenges in early identification and management of individuals at risk of requiring blood transfusion. In some cases, delayed recognition of hemorrhage or insufficient access to timely interventions may exacerbate the need for blood transfusion and increase the risk of adverse maternal outcomes. This underscores the imperative need for the development of effective methodologies aimed at identifying high-risk patients. Predicting PPH before delivery is crucial for enhancing patient outcomes, enabling timely transfer to higher levels of care, advanced preparation, and implementation of prophylactic therapies^8^.

Despite historical studies on risk factors related to PPH, predicting the occurrence of PPH remains challenging. Risk factors such as abnormal placentation, placental abruption, severe preeclampsia, and intrauterine fetal demise have been identified^9^, but predicting a woman's risk of PPH upon labor admission involves incorporating known risk factors and approximating the probability using a risk strata scheme. In addition, a significant portion of PPH cases involve patients lacking known risk factors, presenting a challenge for traditional models that often fall short in predicting such instances^10,11^.

Some studies have developed PPH prediction models based on hemoglobin (Hb) levels and blood transfusion needs. Visual estimates of blood loss are deemed inaccurate^12^, and the gravimetric method for measuring blood loss has been validated in various studies^13^. Hb levels, especially concentrations below 80 g/L, appear to be a more accurate factor for evaluating and predicting PPH^14^.

Current risk-based stratification guidelines endorsed by The American College of Obstetricians and Gynecologists (ACOG) and California Maternal Quality Care Collaborative (CMQCC) utilize decision tree algorithms based on clinical consensus, expert opinion, and prior observational data^15–17^. However, a validated clinical prediction model suitable for deployment on labor and delivery units for PPH is currently lacking^18^. Traditional statistical methods historically formed the basis for risk prediction. However, the current literature indicates a shift towards embracing machine learning (ML) driven by advanced computer algorithms, particularly for individuals lacking conventional risk factors^19^. ML models efficiently automate the processing of non-additive relationships and incorporating complex interaction between factors that otherwise require specialized statistical expertise and time-consuming exploratory data analysis. This holds promising potential for accurately identifying women at the highest risk of PPH, potentially improving obstetric decision-making and clinical outcomes^20–23^. In this study, we attempted to construct an accurate machine-learning model using pre-labor clinical data and basic laboratory measurements to predict postpartum Hb levels in non-complicated singleton pregnancies.

## Methods

This retrospective cohort study was conducted on pregnant women hospitalized in the maternity department of Mahdieh and Arash Hospitals in Tehran, Iran, from February 2016 to October 2019. This study included term pregnant women receiving standard of care and delivering within 24 h with a gestational age of more than 36 weeks and a singleton pregnancy. Exclusion criteria were defined as follows: Patients with Hb decline secondary to trauma, those with hemoglobinopathies, recent smoking, pre-delivery infection, hereditary and acquired coagulopathy and dysregulated coagulation profile (International normalized ratio > 1.5, activated partial thromboplastin time > 35 s) and anticoagulant use, inflammatory and rheumatic diseases, congenital and ischemic heart diseases, familial and congenital liver disease and cirrhosis, ketoacidosis, sepsis, or whole blood and blood product transfusion throughout the study.

Baseline demographics, obstetrics, and laboratory data, including age, gestational age, BMI, gravidity, parity, and abortion history, past medical history, past vaginal or cesarean delivery, labor cause, interval since last pregnancy, placenta location, perioperative blood product transfusion (as exclusion criteria), and baseline blood cell count and serum fibrinogen levels were obtained from patient medical records and paper-based questionnaires. Maternal and neonatal outcomes other than post-delivery maternal Hb levels were documented but not considered for the current study. Placental orientation was determined using ultrasound reports on sessions performed throughout the pregnancy.

Hb levels were routinely checked twice for each pregnant woman admitted to the study center; with the latter used as the main endpoint of the study. Two venous blood samples obtained in supine position were procured from each participant during distinct time intervals. The first sample was drawn within 24 h before the onset of labor to obtain serum and plasma measurements for complete blood count and laboratory measurements, while the second sample was collected 24 h post-delivery to evaluate Hb changes as a surrogate outcome for perioperative delivery and PPH. Samples were collected in vacutainer tubes containing 0.129 mol/L sodium citrate for coagulation assays, platelet count, fibrinogen, and other blood sample characteristics. Cell count was performed using automated hematology analyzers. Fibrinogen levels were assessed using the clot-based functional assay (Clauss method) using standard clinical laboratory kits. The local reference range for fibrinogen was 250-450 mg/dL.

Informed written consent to include anonymized laboratory and clinical patient data was obtained from all participants meeting the inclusion criteria. The proposal for this research has been approved by the Ethics Committee of Infertility and Reproductive Health Research Center (IRHRC), Shahid Beheshti University of Medical Sciences (2014, SBMU, REC 299). Study procedures have been performed in accordance with the Declaration of Helsinki.

### Statistical analysis

Percentages were calculated for categorical variables, whereas mean and standard deviation were calculated for continuous variables. The association between pre-labor variables in the dataset with significant hemodynamic changes throughout the labor was evaluated by comparing the distribution of the aforementioned characteristics in those with or without significant Hb decline (≥ 2.5 g/dL) using independent t-test for continuous variables and chi-square tests for categorical variables as appropriate. Considering the lack of clinical relevance for absolute decline in hemoglobin within the normal range, linear Hb values were prioritized as the outcome of choice. To investigate the extent of association with post-delivery Hb levels, the input of ML models chosen using the feature selection approach described further below were incorporated in a multivariate linear regression model, alongside their crude estimates. All statistical analyses were carried out using Stata version 18.0. Alpha was set a priori at 0.05.

### Data preparation and feature selection

The data was first split randomly into training and test datasets with an 8:2 ratio. To establish a consistent workflow and reproducible and comparable results, a preprocessing and feature selection pipeline was devised before evaluating the predictive accuracy of each included model. Preprocessing and data preparation were carried out by first removing variables with zero or near zero variances in the training dataset. Missing data was then imputed using bagged tree models for each predictor. Continuous data was scaled down between 0 and 1 to improve the predictive accuracy of dependent algorithms. Feature selection was performed using recursive feature selection based on random Forest and elastic net regularization algorithms with tenfold repeated (n = 3) cross-validation on training data by sequentially eliminating variables with least importance. The optimal model predictors for various combinations of independent variables were fitted to the remaining folds to assess the predictive accuracy of the selected model using the root mean squared error (RMSE) measure. The top five sets of predictors, chosen based on lower error rates, were then incorporated into the ML models.

### Algorithm training and validation

To achieve optimal predictive accuracy, a comprehensive evaluation of various ML algorithms for regression output was conducted. The evaluated algorithms included Linear Regression (LR), Support Vector Machine with a linear kernel (SVM; implemented using the e1071 package), Multilayer Perceptron/Artificial Neural Networks with a single hidden layer employing resilient backpropagation and weight backtracking (ANN; implemented using the Neuralnet package). Additionally, Extreme Gradient Boosting with a linear base learner and regularization (XGBM; implemented using the xgboost package and gblinear booster) and Regression Tree (RT; implemented using the rpart package) models were considered.

Hyperparameter tuning and initial model evaluation were performed on the training dataset using a fivefold cross-validation strategy. The optimal hyperparameter values for each model were determined through the grid search approach implemented in the Caret package in R to obtain lowest RMSE. After determining optimized hyperparameters for each algorithm, the models were retrained on the whole training dataset. The accuracy of each model was evaluated using mean absolute error, RMSE, RMSE-standard deviation ratio (RSR), percent bias (PBIAS), and R-squared (R^2^) on validation dataset.

To further enhance the precision of the final model, two meta-ensemble models were developed. These ensembles incorporated the predicted values from the top three performing predictive algorithms from the earlier steps into a Generalized Linear Model (GLM) and a Random Forest algorithm, respectively. The retraining of the model with these meta-ensembles followed the same process as described earlier. The model with the most accuracy was chosen to be utilized in the interactive web platform. Preprocessing and algorithm training was carried out in the R programming language (version 4.3.1) and Caret (Version 6.0) framework^24,25^.

### Interactive platform

An AI interactive platform was created using the RShiny app development platform to provide the most accurate estimation of post-delivery Hb levels. Users can input selected features, which were previously identified, and customize them as input parameters. The platform calculates predicted Hb values along with average 95% prediction intervals derived from the 2.5% and 97.5% percentiles of predictive errors observed in the test subset.

A subsequent one-tailed test will be conducted to examine whether predicted Hb value is comparable to 8g/dL cut point, if so, it is flagged as a high likelihood of requiring post-delivery red blood cell transfusion. Additionally, if the user inputs values outside the range of the training dataset, the platform issues a cautionary warning along with the predicted values, ensuring users are aware of potential limitations in extrapolating predictions beyond the training dataset range. This approach enhances user awareness and promotes cautious interpretation of predictions, contributing to the platform's overall reliability.

## Results

After exclusion of 77 patients, a total of 1974 patient were included in the analyses. The average age (± SD) and BMI of women participating in the study was 27.76 ± 5.76 years and 25.13 ± 3.42 kg/m^2^, respectively. Patients were most likely to deliver past the 37 weeks’ gestation. Hypertension and gestational diabetes mellitus (GDM) were observed in 6.2 and 7.8%, of the participants (Table 1). Average post-delivery hemoglobin was 10.97 ± 1.25 g/dL. Data on delivery type was available on 1966 patient, based on which, 1075 women underwent natural vaginal delivery. Mean duration of labor was 5.09 ± 4.54 h in participants with natural vaginal delivery.

**Table 1:** Baseline characteristics and clinical history and presentation.

History and admission data	Total (n = 1990)	Outcome		*p*-value
		Hb decline < 2.5 g/dL (n = 1794)	Hb decline ≥ 2.5 g/dL (n = 180)	
Age	27.764 (5.755)	27.948 (5.735)	25.945 (5.652)	**< 0.001**
BMI	25.126 (3.423)	25.121 (3.463)	25.171 (3.041)	0.858
Gestational age				
< 36 weeks	127 (6.6%)	124 (7.1%)	3 (1.7%)	**< 0.001**
36 weeks	67 (3.5%)	65 (3.7%)	2 (1.1%)	
37 weeks	219 (11.4%)	205 (11.7%)	14 (8.0%)	
38 weeks	525 (27.3%)	493 (28.2%)	32 (18.2%)	
39 weeks	518 (27.0%)	454 (26.0%)	64 (36.4%)	
40 weeks	377 (19.6%)	324 (18.6%)	53 (30.1%)	
Above 40 weeks	89 (4.6%)	81 (4.6%)	8 (4.5%)	
Past natural vaginal delivery	747 (37.8%)	706 (39.4%)	41 (22.8%)	**< 0.001**
Past cesarean section	467 (23.7%)	450 (25.1%)	17 (9.4%)	**< 0.001**
Hypertension	123 (6.2%)	121 (6.7%)	2 (1.1%)	**0.003**
Migraine	5 (0.3%)	5 (0.3%)	0 (0.0%)	0.478
Seizure/epilepsy	6 (0.3%)	5 (0.3%)	1 (0.6%)	0.520
Impaired glucose tolerance	39 (2.0%)	36 (2.0%)	3 (1.7%)	0.755
Diabetes mellitus	6 (0.3%)	6 (0.3%)	0 (0.0%)	0.437
Gestational diabetes mellitus	155 (7.9%)	144 (8.0%)	11 (6.1%)	0.362
Progesterone vaginal suppository use	562 (28.5%)	529 (29.5%)	33 (18.3%)	**0.002**
Asthma	9 (0.5%)	9 (0.5%)	0 (0.0%)	0.341
Anemia	25 (1.3%)	23 (1.3%)	2 (1.1%)	0.845
Urinary tract infection within pregnancy	40 (2.0%)	39 (2.2%)	1 (0.6%)	0.142
Nephrolithiasis	10 (0.5%)	10 (0.6%)	0 (0.0%)	0.315
Hepatitis B	5 (0.3%)	5 (0.3%)	0 (0.0%)	0.478
Tuberculosis	1 (0.1%)	1 (0.1%)	0 (0.0%)	0.751
Pruritus	9 (0.5%)	9 (0.5%)	0 (0.0%)	0.341

Significant values are in bold.

In crude analyses, age, gestational age, past delivery type, and hypertension were baseline characteristics associated with significant drop in Hb. The use of progesterone vaginal suppository was also linked with hb decline < 2.5 g/dL. Gravidity, parity, cesarean delivery, delivery indications, anterior placenta location, and pre-delivery Hb and platelet were among obstetrics factors linked with 2.5 g/dL decline in secondary Hb (Table 2).

**Table 2:** The association between Pre-labor obstetrics data and hemoglobin drop greater than 2.5g/dL.

Pre-labor obstetrics data	Total (n = 1990)	Outcome		*p*-value
		Hb decline < 2.5 g/dL (n = 1807)	Hb decline ≥ 2.5 g/dL (n = 183)	
Gravidity				
1	745 (37.8%)	636 (35.5%)	109 (60.9%)	**< 0.001**
2	682 (34.6%)	638 (35.6%)	44 (24.6%)	
3	360 (18.3%)	342 (19.1%)	18 (10.1%)	
4	128 (6.5%)	121 (6.8%)	7 (3.9%)	
5	33 (1.7%)	32 (1.8%)	1 (0.6%)	
6	13 (0.7%)	13 (0.7%)	0	
≥ 7	10 (0.5%)	10 (0.6%)	0	
Parity				
0	866 (43.9%)	738 (41.2%)	128 (71.1%)	**< 0.001**
1	738 (37.4%)	703 (39.2%)	35 (19.4%)	
2	298 (15.1%)	284 (15.8%)	14 (7.8%)	
3	50 (2.5%)	48 (2.7%)	2 (1.1%)	
4	10 (0.5%)	9 (0.5%)	1 (0.6%)	
≥ 5	10 (0.5%)	10 (0.5%)	0	
Placenta orientation				
Anterior	1,001 (50.7%)	895 (49.9%)	106 (58.9%)	**0.021**
Posterior	568 (28.8%)	526 (29.3%)	42 (23.3%)	0.091
Fundal	284 (14.4%)	260 (14.5%)	24 (13.3%)	0.673
Lateral	136 (6.9%)	122 (6.8%)	14 (7.8%)	0.622
Placenta previa	2 (0.1%)	2(0.1%)	0	0.648
Delivery indication				
Labor pain	1400 (70.9%)	1277 (71.2%)	123 (68.3%)	0.422
Rupture of membrane	469 (23.8%)	413 (23.0%)	56 (31.1%)	**0.015**
Reduced fetal movement	143 (7.2%)	129 (7.2%)	14 (7.8%)	0.772
Post due date	52 (2.6%)	50 (2.8%)	2 (1.1%)	0.181
Pregnancy-induced hypertension	8 (0.4%)	8 (0.4%)	0	0.369
Repeated Cesarean section	355 (18.0%)	345 (19.2%)	10 (5.6%)	**< 0.001**
Intrauterine growth restriction	15 (0.8%)	15 (0.8%)	0	0.218
Fetal demise	161 (8.2%)	154 (8.58%)	7 (3.9%)	**0.028**
Pre-labor hemoglobin level (g/dL)	12.12 (1.26)	12.04 (1.21)	13.09 (1.26)	**< 0.001**
Pre-labor platelet count (per 1000μL)	219.88 (63.35)	219.81 (63.11)	220.66 (65.97)	0.887
Serum fibrinogen (mg/dL)	268.31 (55.79)	274.78 (52.34)	203.86 (47.60)	** < 0.001**
Delivery type				
Natural vaginal delivery	1,066 (54.2%)	987 (55.3%)	79 (43.9%)	**0.004**
Cesarean section	900 (45.8%)	799 (44.7%)	101 (56.1%)	

Significant values are in bold.

### Variable selection and model training

**Figure 1:**
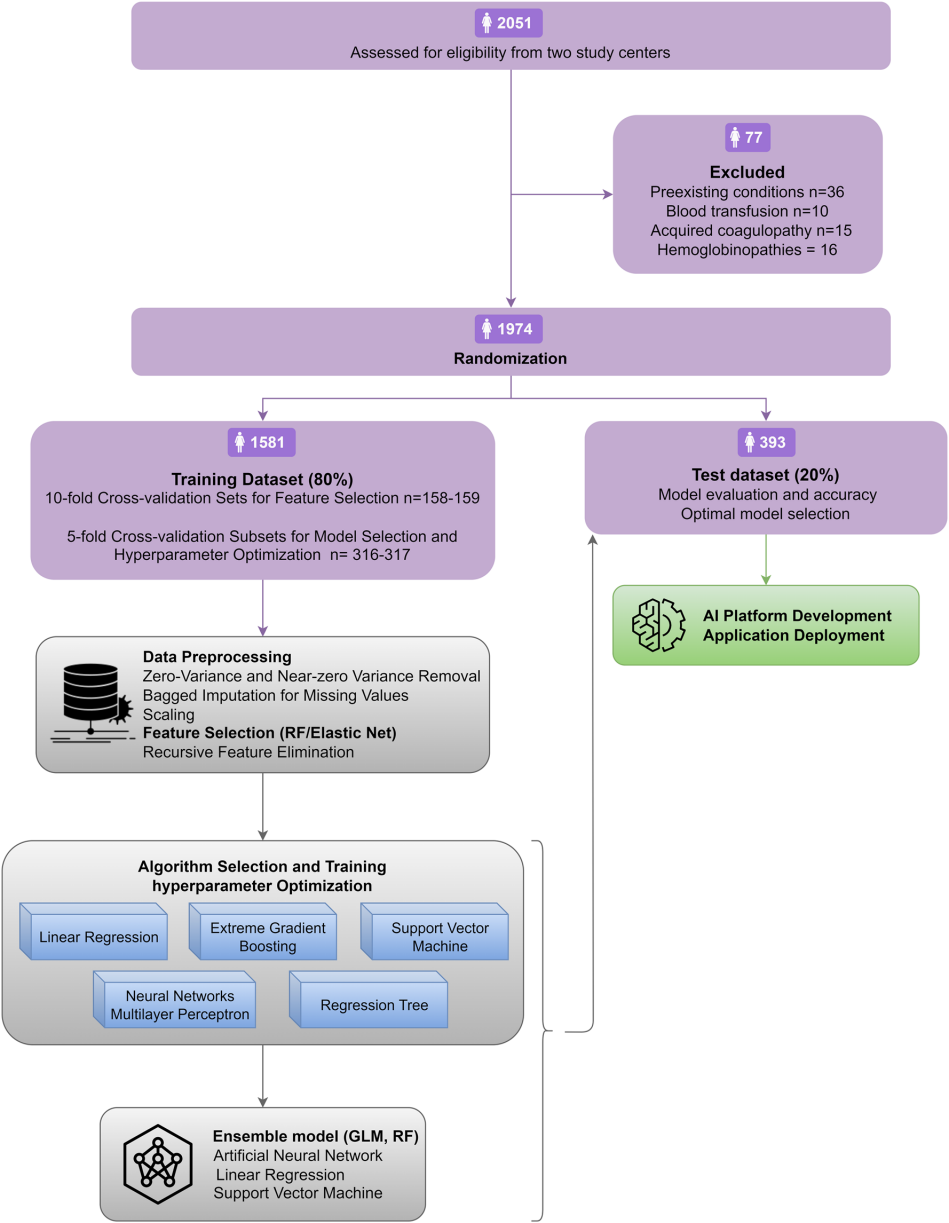
Flow diagram of included participants and overall workflow of the study.

The overall workflow of the current study is illustrated in Fig. 1. The initial phase of preprocessing involved the removal of variables with zero and non-zero variance towards the study outcome. The remaining variables to be assessed for subsequent stage were as follows: age, BMI, gravidity, parity, gestational age, placental orientation, delivery indication, primary (pre-labor) Hb, primary platelet count, serum fibrinogen, GDM, hypertension, suppository progesterone use throughout the pregnancy.

**Figure 2:**
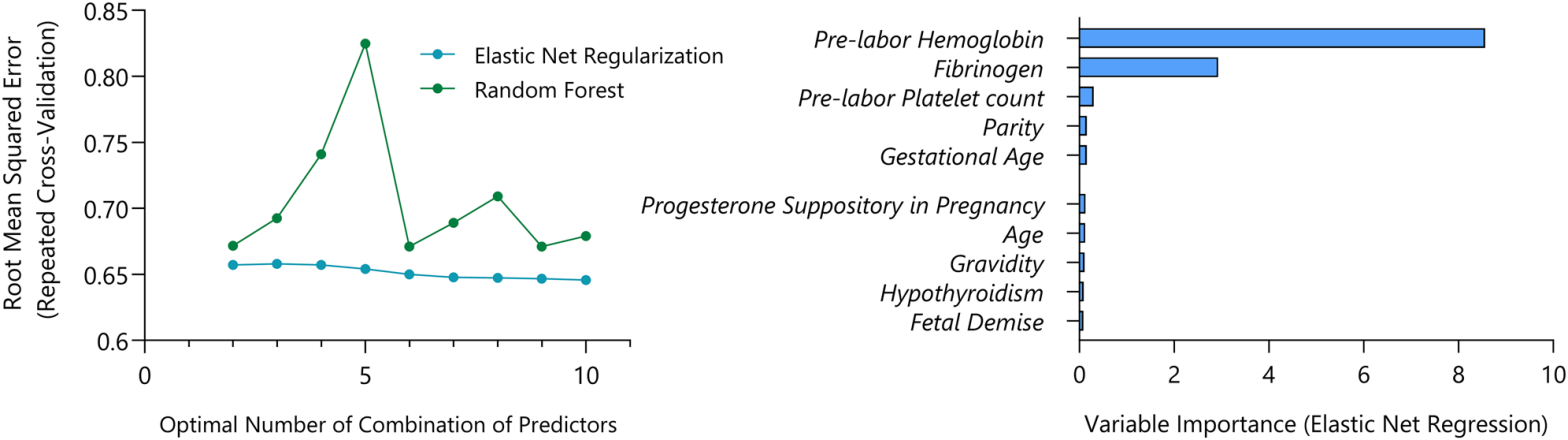
Feature selection and variable importance investigated using Random Forest and Elastic Net Regression. The top 5 predictors identified in the Elastic Net model were chosen as input for all subsequent machine learning models.

Following comprehensive preprocessing and data splitting, the variables in the training dataset were further examined in the recursive feature elimination stage by evaluating their predictive accuracy, in conjunction with other variables, to determine the more accurate combination of predictors explaining the secondary Hb level variance. The top 5 predictors identified using the elastic net regression were as follows: primary Hb, serum fibrinogen, gestational age, primary platelet count, and parity. These selected predictors constitute the final set employed for algorithm training (Fig. 2). Interestingly, while cesarean section was associated with significant drop in Hb, the type of delivery was not correlated to linear changes in post-delivery Hb level.

Multivariate regression model revealed that increasing gestational age is associated with a marginal increase in secondary hemoglobin (− 0.31g/dL reduction in secondary Hb in 40 weeks compared 36 weeks gestation). Expectedly, pre-delivery laboratory values including primary Hb, platelet count, and serum fibrinogen were linked with higher secondary Hb. Moreover, increasing parity was associated with higher post-delivery Hb levels (Table 3).

**Table 3:** Multivariate model demonstrating the top variables associated with post-delivery hemoglobin identified using elastic net regression feature selection.

Predictors	Multivariate model			Crude association*	
	B (95% CI)	*p*-value	Variance inflation factor	B (95% CI)	*p*-value
Parity	0.09 (0.05 to 0.12)	** < 0.001**	1.03	0.06 (0.00–0.12)	***0.054***
Gestational age at labor (vs. 36 weeks)	Ref	–	–	Ref	–
Below 36 weeks	0.05 (− 0.14 to 0.25)	0.589	2.76	− 0.29 (− 0.66 to 0.07)	0.116
37 weeks	− 0.17 (0.35 to 0.01)	***0.065***	3.89	− 0.36 (− 0.49 to 0.15)	**0.034**
38 weeks	− 0.16 (− 0.33 to 0.01)	***0.068***	6.61	− 0.30 (− 0.62 to 0.01)	***0.059***
39 weeks	− 0.25 (− 0.42 to − 0.08)	**0.003**	6.59	− 0.27 (− 0.59 to 0.04)	0.089
40 weeks	− 0.31 (− 0.48 to − 0.13)	**0.001**	5.51	− 0.17 (− 0.49 to 0.15)	0.306
Above 40 weeks	− 0.23 (− 0.44 to − 0.02)	**0.031**	2.27	− 0.35 (− 0.75 to 0.04)	0.076
Pre-labor hemoglobin level	0.83 (0.80 to 0.85)	**< 0.001**	1.10	0.71 (0.67 to 0.74)	** < 0.001**
Pre-labor platelet count (per 1000/μL)	0.77 (0.30 to 1.23)	**0.001**	1.01	1.93 (1.07 to 2.80)	** < 0.001**
Serum fibrinogen (per 100 mg/dL)	1.05 (1.00 to 1.11)	**< 0.001**	1.07	0.62 (0.52 to 0.71)	** < 0.001**

^#^Multiple linear regression model; variable selection was carried out by including the pre-labor predictors used for ML models. *Simple linear regression model.

Significant and near-significant values are highlighted in bold and bolditalics.

Next, we constructed 5 ML models based on the selected variables and the algorithms visualized in Fig. 1. The optimal hyperparameters for SVM were epsilon-type regression with 0.25 constant of regularization term. Regression tree was fit with a complexity parameter of 0.003. XGBM was trained using 50 max boosting iterations and 0.1 L2 regularization term on weights. No interaction constraints were imposed. The neural network used in this work was a simple fully-connected network with one hidden layer with three nodes and sigmoid activation function and a single output node with linear activation function. The ensemble models utilized the results of the top predictive algorithms in meta generalized linear and random forest models.

### Validation and deployment

**Figure 3:**
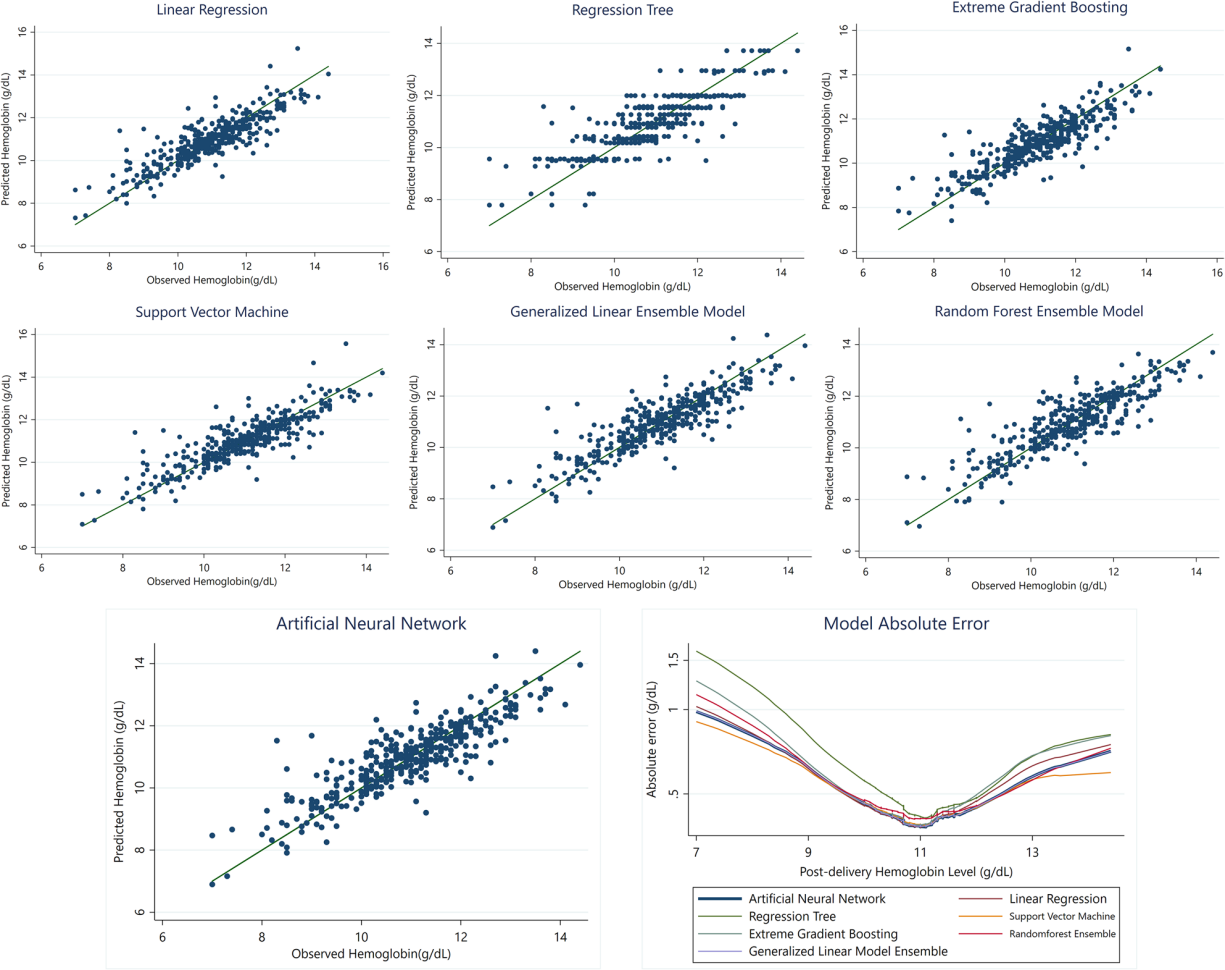
Predictive accuracy and error of all evaluated algorithms. Artificial neural network/Multilayer perceptron had a marginal improvement over other included models. Expectedly, predictive accuracy was lower within lower and upper bounds of post-delivery hemoglobin.

The accuracy of models is provided in detailed in Table 4. All models performed with acceptable predictive precision. Nevertheless, the use of ANN was associated with marginally improved results compared to other models (Fig. 3). Training time was the longest for XGBM followed by ANN, and SVM due to a greater number of tunable hyperparameters. Utilizing ensemble models did not result in a considerably improved fit to observed values compared to ANN and were not taken into consideration. The mean absolute error of the final model was 0.622 g/dL, indicating high accuracy of the model in predicting post-delivery Hb level. The RSR for this model was close to 0.5, which provides a standardized measure that further confirms the high precision of the model. The model was deployed in https://predictivecalculators.shinyapps.io/ANN-HB/ as a standalone interactive webpage, which calculates the predicted value along with the 95% prediction intervals as the measure of model uncertainty.

**Table 4:** Performance of the algorithms and models used for predicting post-delivery hemoglobin level.

Metrics	Algorithms					Stack models	
	LR	RT	SVM	XGBM	ANN	GLM	RF
MAE	0.4575	0.5179	0.4491	0.4747	**0.4502**	0.4508	0.4645
RMSE	0.6312	0.7105	0.6349	0.6487	**0.6217**	0.6222	0.6407
PBIAS	**− 0.0646**	− 0.6026	**− **0.4554	0.0963	− 0.0775	− 0.0767	− 0.0932
R^2^	0.7521	0.6860	0.7466	0.7382	**0.7596**	0.7592	0.7446
RSR	0.5133	0.5777	0.5162	0.5275	**0.5055**	0.5059	0.5210

Bold values highlight the most optimal model based on the performance measure of the corresponding row.

## Discussion

This investigation was undertaken utilizing an extensive dataset procured from two tertiary care hospitals with the aim of constructing a predictive model for postpartum hemoglobin (Hb) levels subsequent to childbirth. The dataset originally encompassed a multitude of variables, acquired both prenatally and postnatally, in the context of both normal vaginal delivery (NVD) and cesarean section (CS). All variables assessed either prior to or early within labor were examined for predictive value using both a statistical and a semi-automated workflow displayed throughout the work.

Consistent with previous studies^26,27^, there was a positive correlation between primary and secondary Hb values, as well as serum fibrinogen levels and platelet count with secondary Hb. Fibrinogen plays a crucial role in the coagulation cascade and serves as the central element in clot formation. During pregnancy, fibrinogen levels experience a gradual rise of up to 50%, correlating with advancing gestational age and reaching their peak in the third trimester. This elevation in fibrinogen levels is a fundamental aspect of the coagulation system's adaptive response, strategically aimed at mitigating the potential risks of adverse hemorrhagic outcomes during pregnancy^28^. Since evaluating fibrinogen level is characterized by expeditiousness, simplicity, and cost-effectiveness, it can be easily used before labor for predicting hematological outcomes after delivery. Anticipating transfusion needs based on pre-labor fibrinogen levels enables healthcare facilities to optimize resource allocation. This includes ensuring the availability of blood products and skilled medical staff, leading to more efficient and cost-effective maternal healthcare delivery.

Among variables associated with both linear and categorical hemoglobin outcome, gestational age emerged as a significant variable exhibiting a pronounced association with the decline in hemoglobin levels post-delivery. Consistent with our findings, analogous results have been reported in other studies, collectively suggesting that patients with a later gestational age at delivery are at an elevated risk of postpartum hemorrhage^29^. The association between gravidity and parity and a diminished likelihood of hemoglobin decline following delivery is also not unexpected considering the elevated risk of PPH among nulliparous women^30^. Notably, our investigation also demonstrated that the utilization of vaginal suppositories containing Progesterone is associated with a reduction in postpartum hemorrhage. This phenomenon is hypothesized to be attributed to the modulatory influence of progesterone on myometrial contractility, resulting in enhanced uterine contraction and subsequently diminished postpartum bleeding^31^.

Given the substantial global impact of PPH on maternal mortality, there is a pivotal need to delineate a predictive variable for hemorrhage and associated volume loss^32,33^. A universally accepted definition of postpartum hemorrhage (PPH) remains elusive, as multiple definitions are presently employed globally^34,35^. Although common definitions facilitate cross-country comparisons of PPH incidence rates, the clinical significance of quantified blood loss in otherwise robust and healthy parturient women is subject to skepticism^32,36^. Assessing the prevalence of clinically severe hemorrhage may prove more pertinent, taking into account the rate, total volume of blood loss, need of transfusion, post-delivery Hb and the efficacy of therapeutic interventions^37^. The gravity of PPH is contingent upon the maternal response to treatment, the pace and magnitude of blood loss, and the overall health of the patient, including pre-existing conditions which renders individuals more susceptible to decompensation in the presence of peripartum bleeding. Predicting the post-delivery Hb would play an essential role in clinical management of postpartum hemorrhage, volume loss and transfusion need. ML algorithms bring unprecedented analytical capabilities to the realm of maternal healthcare that may be specifically trained for this task. By processing vast datasets encompassing diverse patient profiles, ML models can discern intricate patterns and relationships within the data and may achieve higher predictive accuracy than experts within the same field.

## Conclusion

This work demonstrated that both ML and statistical models demonstrate a high level of accuracy in predicting Hb level within 24 h post-delivery based on data accessible upon admission for labor including fibrinogen level. This study employs an analytical approach that has not been extensively explored or applied in obstetrics. However, it is crucial to note that this “proof of concept” must undergo prospective testing in larger population-based studies with heterogeneous sample sizes to validate its effectiveness. Despite the aforementioned factors, this study was limited by the omission of cases with allogenic blood product transfusion and other laboratory coagulation factors which could have given a more comprehensive profile and potentially improved the predictive accuracy of the model. While, the removal of non-complicated cases, traumatic cases, coagulopathies, and rare occurrences of factors strongly associated with intrapartum blood loss and PPH such as placental abruption in this study provided a more uniform distribution of participants, this choice may limit the generalizability of the results and preclude the predictive capability of the models for a general and geographically-distinct population. Overall, the results underscore the potential of machine learning methodologies to enhance clinical prediction and optimizing patient outcomes in the field of obstetrics.

## Data Availability

The dataset analyzed during the current study available from the corresponding author on reasonable request and with permission of the research and ethics committee of the study centers.
